# Climatic niche differences among *Zootoca vivipara* clades with different parity modes: implications for the evolution and maintenance of viviparity

**DOI:** 10.1186/s12983-021-00403-2

**Published:** 2021-06-29

**Authors:** J. L. Horreo, A. Jiménez-Valverde, P. S. Fitze

**Affiliations:** 1 Department of Genetics, Physiology and Microbiology, Complutense University of Madrid, C/José Antonio Novais 12, 28040 Madrid, Spain; 2 Department of Biodiversity and Evolutionary Biology, Museo Nacional de Ciencias Naturales (MNCN-CSIC), José Gutiérrez Abascal 2, 28006 Madrid, Spain; 3 Universidad de Alcalá, Departamento de Ciencias de la Vida, Grupo de Investigación de Biología del Suelo y de los Ecosistemas Subterráneos, A.P. 20 Campus Universitario, 28805 Alcalá de Henares, Madrid, Spain

**Keywords:** Cold climate hypothesis, Parity mode evolution, Maternal manipulation hypothesis, Ecological niche, Oviparity, Viviparity

## Abstract

Parity mode (oviparity/viviparity) importantly affects the ecology, morphology, physiology, biogeography and evolution of organisms. The main hypotheses explaining the evolution and maintenance of viviparity are based on bioclimatic predictions and also state that the benefits of viviparity arise during the reproductive period. We identify the main climatic variables discriminating between viviparous and oviparous Eurasian common lizard (*Zootoca vivipara*) occurrence records during the reproductive period and over the entire year.

Analyses based on the climates during the reproductive period show that viviparous clades inhabit sites with less variable temperature and precipitation. On the contrary, analyses based on the annual climates show that viviparous clades inhabit sites with more variable temperatures.

Results from models using climates during reproduction are in line with the “selfish-mother hypothesis”, which can explain the success of viviparity, the maintenance of the two reproductive modes, and why viviparous individuals cannot colonize sites inhabited by oviparous ones (and vice versa). They suggest that during the reproductive period viviparity has an adaptive advantage over oviparity in less risky habitats thanks to the selfish behaviour of the mothers. Moreover, the results from both analyses stress that hypotheses about the evolution and maintenance of viviparity need to be tested during the reproductive period.

## Background

Reproductive mode (e.g. oviparity, viviparity) is an important biological trait that may affect the ecology, morphology, physiology, biogeography and evolution of organisms [1–3]. While the embryonic development of many oviparous organisms mainly depends on environmental conditions, those of viviparous organisms can at least partially be controlled by mothers. For example, viviparous mothers can control intra-uterine temperature by means of thermoregulation and habitat choice [4], and they provide embrios with nutrients during their development [5]. Two main hypotheses have initially been put forward to explain the evolution and maintenance of viviparity in reptiles. The “cold climate hypothesis” (CCH [6]) advocates that viviparity is an adaptation to cold environments because it allows mothers to raise embryonic temperature above the environmental temperature, what is especially advantageous if environmental temperatures are not high enough for the development and survival of embryos. The “maternal manipulation hypothesis” (MMH) advocates that the main advantage of viviparity resides on its positive effect on offspring viability and development in less favourable environmental conditions [7, 8], which include low and high ambient temperatures, and other unfavourable aspects relevant to embryonic development [9], such as highly variable and unpredictable environments [10, 11].

Both hypotheses exclusively explain the maintenance and evolution of viviparity by a positive effect on offspring fitness. However, selection favours strategies that increase an individuals’ rather than its offspring’s fitness [12, 13]. Thus, adaptations which are positive for mothers should be favoured, regardless of how these adaptations affect the offspring’s fitness [14, 15]. More specifically, selection can increase a mother’s fitness at the expense of her offspring’s fitness, because a mother’s fitness depends on her lifespan, and on how she resolves the trade-offs between survival and reproduction and between the mother’s and the offspring’s optimal maternal investment [13, 14, 16, 17]. Based on these ideas, a novel hypothesis, the “selfish-mother hypothesis” (SMH), may explain the evolution and maintenance of viviparity.

The SSH predicts that females favour their own lifetime reproductive success and survival, rather than her offspring’s sucess [18]. Because gravidity of viviparous females lasts much longer than that of oviparous females [19, 20], viviparous females in risky habitats (e.g., habitats that prolongate gravidity) are more likely exposed to a trade-off between favouring their survival and fitness over that of her offspring, resulting in offspring of lower viability and in extreme cases abortion of the eggs [21]. The hypothesis further predicts that in less risky habitats viviparous females are not exposed to this trade-off and that they produce more offspring of higher viability than oviparous females, which may favour the evolution and maintenance of viviparity.

Despite the differences among the three hypotheses, they all predict that viviparity is an adaptation to the environmental conditions prevailing during the reproductive period, rather than those prevailing during the entire year (e.g. [22, 23]). Studies addressing the maintenance and evolution of viviparity from a biogeographic or macroecological perspective and including taxa belonging to one [23, 24] or many families [22, 25], mostly provided evidence in favour of the CCH hypothesis. However, several observations are not congruent with the CCH: first, viviparity is relatively common in tropical snakes and thus it is not restricted to species inhabiting the temperate zone [26]; and second, the early evolution of viviparity in squamates happened during the Jurassic, i.e., during a warmer period [2].

Moreover, several studies highlighted that current distributions may not reflect the climate at the time and place where viviparity evolved [6, 22]. This suggests that current data may unravel parameters explaining the maintenance of viviparity, but not necessarily those explaining its evolution. In other words, the more time passed since the evolution of viviparity, the lower will be the likelihood that current climatic and distributional data allow to infer the drivers of its evolution. More specifically, the drivers of events that happened tens of millions of years ago may be less likely to pin down (e.g. [23, 24]) and Pyron and Burbrink [27] suggested that the use of detailed species-level data and taxa with more recently evolved viviparity may increase the likelihood of detecting the drivers of their evolution. Moreover, species-level data may avoid biases inherent to studies including many different species [27]. Thus, species with bimodal reproduction might be good study candidates [25].

**Fig. 1:**
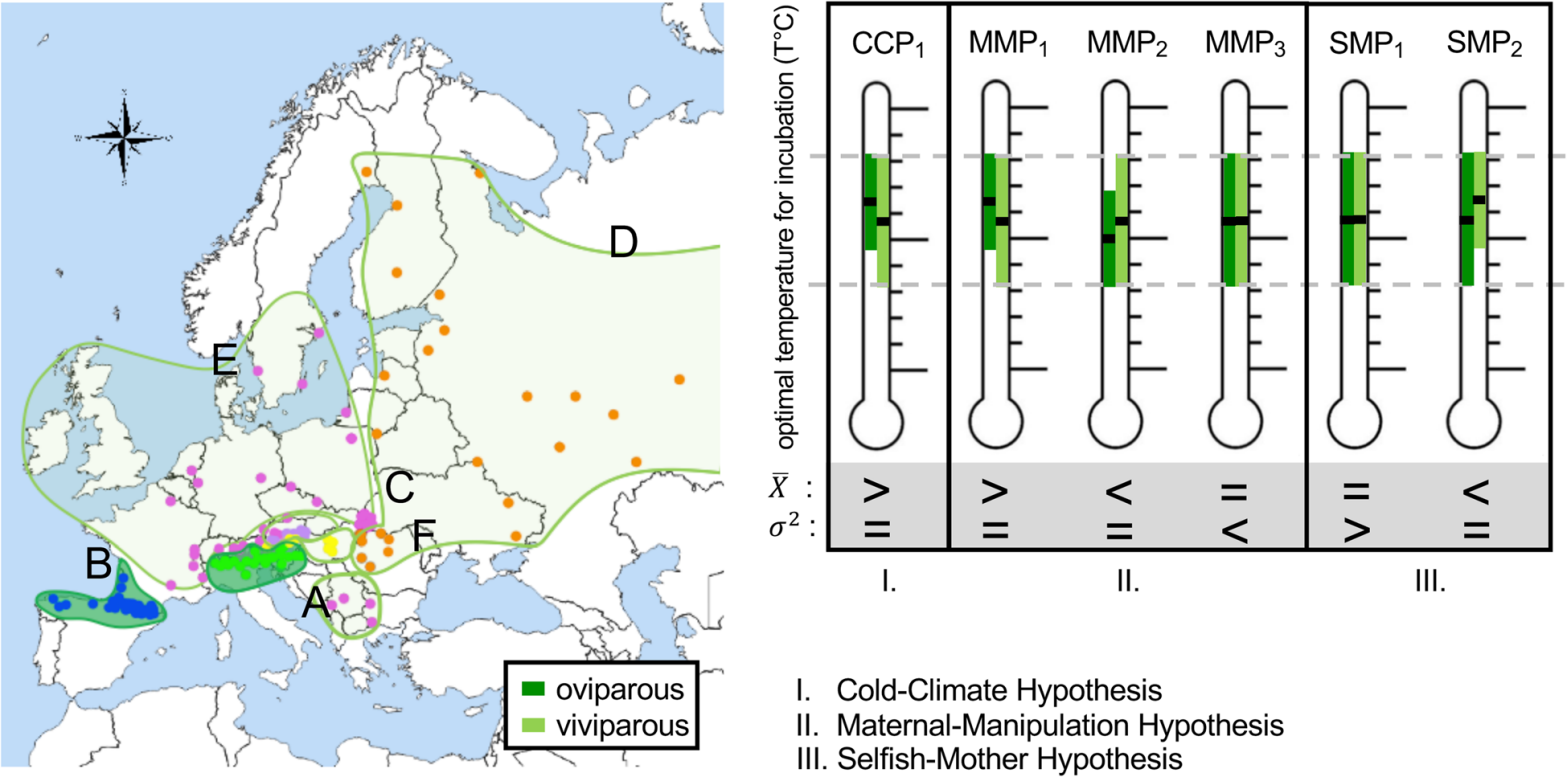
Schematic distribution of the *Zootoca vivipara* clades in Eurasia and climatic predictions derived from the three hypothesis explaining the evolution of viviparity. Dots on the map refer to populations used to disentangle among the hypotheses and dot colour to the genetic clade affiliation; green: clade A, blue: clade B, purple: clade C, orange: clade D, pink: clade E, and yellow: clade F. Distributions of viviparous clades are coloured in light green and those of oviparous lizards are in dark green. Letters on the geographic map refer to genetic clades [29]. On the right of the map, climatic predictions (abbreviated as ‘P’), derived from three hypothesis explaining the evolution and maintenance of viviparity. Predicted range differences of optimal incubation temperature and differences in average incubation temperature (black ‘-‘within range) for viviparous (light green) and oviparous (dark green) lizards are shown for each hypotheses. $\overline{X}$ corresponds to average incubation temperature, *σ*^2^ to the variance in incubation temperature, ‘>’ indicates that values are bigger in oviparous clades, ‘=’ indicates that there exists no differences among parity modes, and ‘<’ indicates bigger values in viviparous clades. The predictions for each of the three hypotheses are abbreviated using lowercase numbers. For simplicity, only the predictions related to temperature are shown, while the general predictions are stated in the article’s text

Here we tested for differences in the inhabited climatic niche among oviparous and viviparous clades of the Eurasian common lizard *Zootoca vivipara* (Lichtenstein, 1823). *Zootoca vivipara* is a small lizard species with bimodal reproductive mode that exhibits the largest geographical and the northernmost distribution of terrestrial reptiles [28]. It consists of six genetic clades (Fig. 1), that evolved in the last ≤4.4 million years [29] and thus more recently than the transitions to viviparity considered in previous studies (in Lambert and Wiens [23], Pincheira-Donoso et al. [24], Watson et al. [25], and Feldman et al. [22], most of the analysed transitions from oviparity to viviparity happened more than 8 Mya). To understand which hypothesis best explains the current distribution, we used fine-scale distributional data of *Z. vivipara*, niche comparison tests, and discrimination models of parity mode (oviparous versus viviparous) based on climatic data. According to the CCH [6], we predicted that on average viviparous lizards inhabit locations with colder average or minimum temperatures during reproduction (Table 1; Fig. 1- prediction CCP_1_). According to the MMH [9], we predicted that on average viviparous lizards inhabit locations with less favourable, i.e. colder (Fig. 1- prediction MMP_1_) or warmer locations (Fig. 1- prediction MMP_2_ [9]), and/or more variable climates (e.g., more variable in temperature and precipitation) than oviparous populations (Table 1; Fig. 1- prediction MMP_3_). According to the SMH, we predicted that viviparous females inhabit locations in which they are less likely exposed to a trade-off between their own survival and fitness, and that of their offspring. Because viviparous *Z. vivipara* females take almost twice as long to lay their eggs (about one month more, for an additional 6–8 embryonic developmental stages [19, 20]) and because gestion of viviparous *Z. vivipara* females is not facultative [19], we predicted that viviparous *Z. vivipara* inhabit locations with lower climatic variability (Table 1; Fig. 1- prediction SMP_1_), in which embryonic development is faster and egg laying is earlier [10, 11]. The shorter gravidity period reduces the time during which the developing eggs negatively affect a female’s locomotion, which reduces a female’s predation risk (e.g, [30, 31]). Moreover, females are also less likely to invest a lot of energy into reproduction without success. According to the SMH, we also predicted that on average viviparous females may live in warmer climates, where embryonic development is faster (Table 1; Fig. 1- prediction SMP_2_ [32, 33]). Finally, if the annual climate reflects the climate prevailing during reproduction, we predicted that differences in climatic niches among viviparous and oviparous populations during the reproductive period will be similar to those based on annual values [22].

**Table 1:** Combinations of differences between parity modes in variables describing variability (second column) and average temperature (third column) and their support for a given hypothesis explaining the evolution of viviparity (Fig. 1). Abbreviations: ↑vivi: viviparous > oviparous, ↓vivi: viviparous < oviparous, ‘-‘: no differences, CCH: cold-climate hypothesis, MMH: maternal manipulation hypothesis, SMH: selfish mother hypothesis, letters and numbers in brackets correspond to the predictions listed in Fig. 1 (e.g. MMP_1_: Manternal Manipulation Prediction 1)

combination Nr.	climatic differences among parity modes		supported hypotheses^a^
	variables describing *variability*	variables describing *average temperature*	
1	–	–	none
2	–	↓vivi	CCH (CCP_1_), MMH (MMP_1_)
3	–	↑vivi	MMH (MMP_2_), SMH (SMP_2_)
4	↑vivi	–	MMH (MMP_3_)
5	↑vivi	↓vivi	MMH (MMP_1,3_)
6	↑vivi	↑vivi	MMH (MMP_2,3_), SMH (SMP_2_)^b^
7	↓vivi	–	SMH (SMP_1_)
8	↓vivi	↓vivi	SMH (SMP_1_)^c^
9	↓vivi	↑vivi	SMH (SMP_1,2_)

^a^ if a given hypothesis makes predictions on average temperature, but not on variability (e.g. the CCH), then combinations including effects on averages and variances (e.g., combinations 5, 6, 8, 9) do not support this hypothesis, given that the hypothesis cannot explain why the differences in variances exist

^b^ if the negative effect of higher variability is smaller than the positive effect of increased temperature

^c^ if the negative effect of lower temperature is smaller than the positive effect of lower variability

## Results

**Fig. 2:**
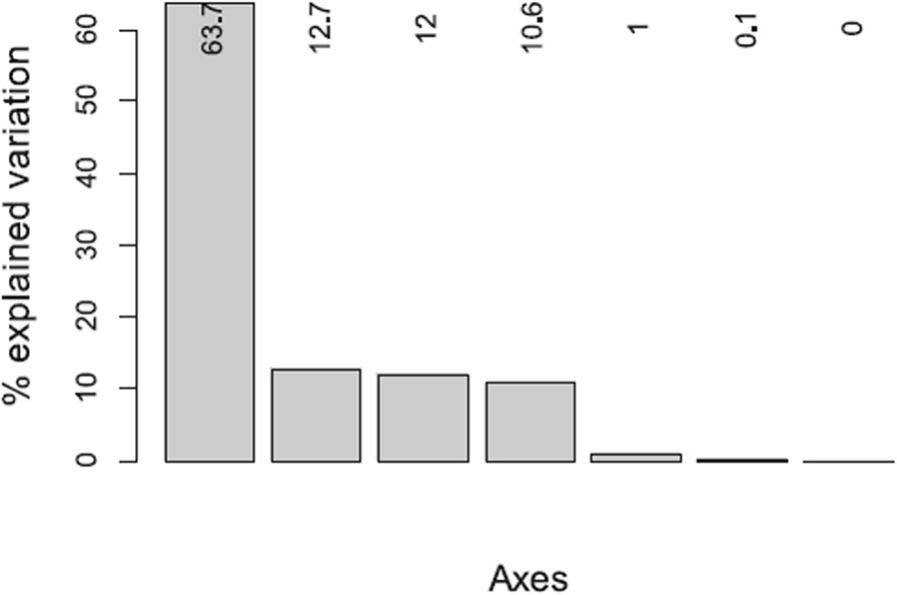
Percentage of variation explained by the PCA axes for the reproductive period

**Fig. 3:**
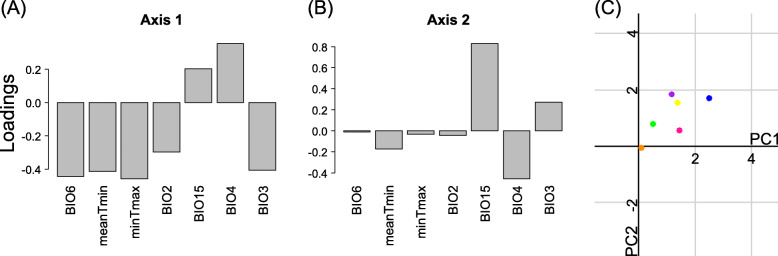
Results of the principal component analysis (PCA) carried out on the climatic variables measured during the reproductive period. Loadings of the climatic variables (for details about the climatic variables see Appendix S1) in the first (**a**: PC1) and second (**b**: PC2) PCA-axis. **c** Ordination space delimited by the two first principal component axes (PC1 and PC2) with centroids of each clade. Green: clade A, blue: clade B, purple: clade C, orange: clade D, pink: clade E, and yellow: clade F

During the reproductive period, principal component analyses of the climatic variables rendered two axes, which accounted for 76.4% of the variation (Fig. 2). The first axis (PC1, 63.7% of the variation explained) was positively related with the seasonality of temperature and precipitation (BIO4 and BIO15, Fig. 3a, for the meaning of the abbreviations of the bioclimatic variables see Supporting Information Appendix S1, S2), and negatively with the other five variables (Fig. 3a). Precipitation seasonality (BIO15) exhibited the lowest loading, while all other variables showed medium loadings (Fig. 3a). The second axis (PC2) explained 12.7% of the variation (Fig. 2), and it mainly represents precipitation seasonality (BIO15) and, to a lesser extent, temperature seasonality (BIO4; Fig. 3b). On average, clade B occupies the coldest areas and areas with the highest seasonal and the lowest diurnal climatic variability, whereas clade D inhabits the warmest areas with the lowest seasonal and the highest diurnal climatic variability (Fig. 3c).

Niche similarity tests showed that C-F exhibit significantly higher *D*_*obs*_ than expected by chance (Table 2), and a relatively high overlap (*D*_*obs*_ = 0.655 [34]). In half of the comparisons, clades exhibited significant non-equivalent niches (Table 2).

**Table 2:** Results of the niche similarity and equivalency tests for the reproductive period. Below the diagonal, pair-wise niche similarity (*D*_*obs*_) scores are given and asterisks indicate a significant two-tailed niche similary test (* *p* < 0.05; in the significant pair, *D*_*obs*_ was higher than expected by chance). Above the diagonal, significances of pair-wise equivalency tests are shown (* *p* ≤ 0.05, ** *p* ≤ 0.01, NS: not significant; in all significant pairs, *D*_*obs*_ was lower than expected by chance)

Subclade	A	B	C	D	E	F
A	–	*	NS	*	NS	NS
B	0.253	–	*	**	**	NS
C	0.275	0.283	–	**	NS	NS
D	0.180	0.045	0.028	–	**	**
E	0.301	0.324	0.366	0.104	–	NS
F	0.388	0.494	0.665*	0.049	0.488	–

**Fig. 4:**
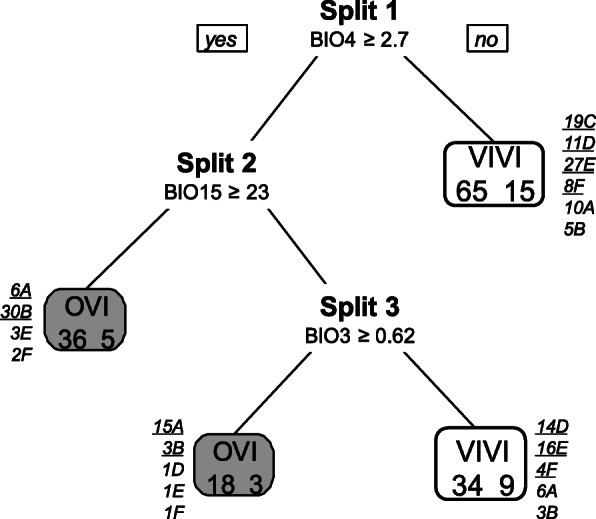
Classification tree for the reproductive period resulting from recursive partitioning with parity mode (oviparous vs. viviparous) as response variable and the seven climatic covariates (Supporting Information Appendix S1) as predictors. In each split, the variable that decreased the impurity the most is indicated and its threshold value is given (BIO4, units: degrees Celsius; BIO15, units: millimetres; BIO3, units: percent). Grey leaves represent the cases classified as oviparous and white leaves represent those classified as viviparous. Numbers within leaves correspond to the number of correctly and incorrectly classified cases (left and right values, respectively). Numbers next to each leave, refer to the number of correctly (underlined) and wrongly classified (not underlined) cases per clade

**Fig. 5:**
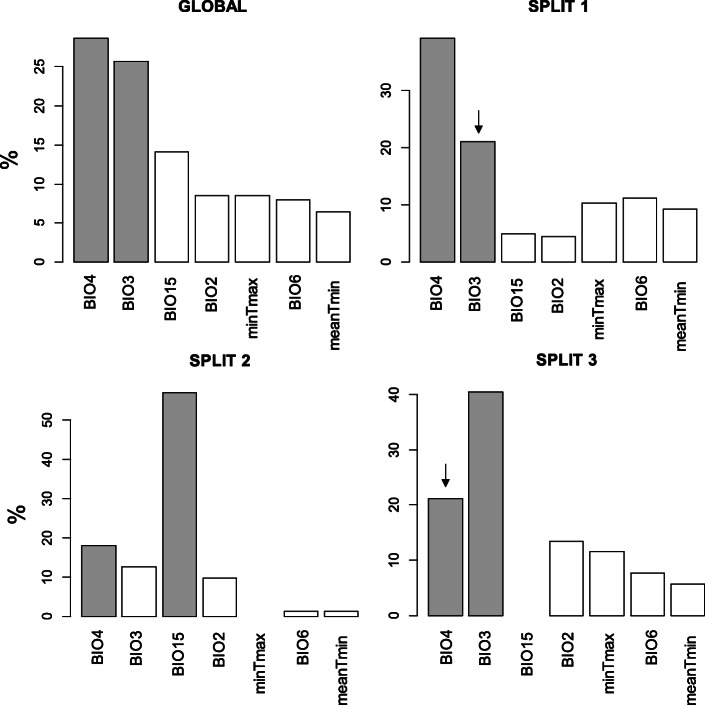
Importance (% of improvement in deviance; y-axis) of the climatic variables (x-axis; for details on their meaning see Appendix S1) in the global classification tree (panel **a**) and in the splits 1–3 of Fig. 4 (panel **b**-**d**). Variables whose effect is larger than expected by chance, i.e., those whose importance is larger than 100/(number of variables), are indicated by grey shaded bars. Arrows above significant bars indicate that the climatic variable was negatively correlated with the main variable, i.e., the variable explaining the highest % of the variance. That is, if the main variable’s effect was negative, the effect of the variable indicated with an arrow was positive, or vice versa

Cross-validation points to a tree of four leaves (three splits) and a correct classification rate of 82.7% (Fig. 4). 54 out of 78 oviparous records (69.2%) and 99 out of 107 viviparous records (92.5%) were correctly predicted. 21 out of 37 occurrences of clade A (56.7%), 33 out of 41 occurrences of clade B (80.5%), 25 out of 26 occurrences of clade D (96.1%), 43 out of 47 occurrences of clade E (91.5%), 12 out of 15 occurrences of clade F (80%), and 100% of the occurrences of clade C were correctly classified (Fig. 4). Globally, variability related variables were most influential in the classification tree (Fig. 5), namely temperature seasonality (BIO4) and isothermality (BIO3). BIO4 was important in all splits, BIO3 was important in all but one (split 2, Fig. 5), and precipitation seasonality (BIO15) played the major role in split 2. In each split, lower values of the main variable were associated with viviparity (Fig. 4). In split 1, viviparity was associated with lower temperature seasonality and higher relative diurnal variability (BIO3, isothermality). In split 2, viviparity was associated with lower seasonality in precipitation and temperature, and in split 3 with lower relative diurnal variability and higher temperature seasonality (Fig. 5). In each split, the most important variable (BIO4 in split 1, BIO15 in split 2, and BIO3 in split 3) was twice as important as the second most relevant variable (Fig. 5), indicating that viviparous populations occupy habitats with lower temperature and precipitation seasonality, and lower isothermality compared to oviparous populations (Fig. 5).

**Fig. 6:**
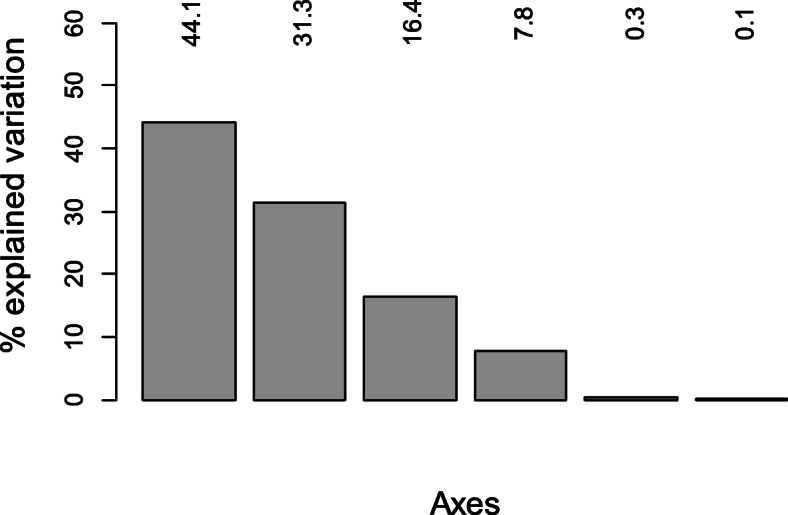
Percentage of variation explained by the PCA axes for the annual period

**Fig. 7:**
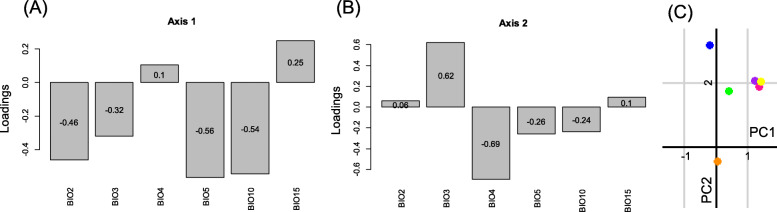
Results of the principal component analysis (PCA) for the annual period. Loadings of the climatic variables (for details about the climatic variables see Appendix S1) in the first (**a**) and second (**b**) PCA-axis. **c** Ordination space delimited by the two first principal component axes (PC1 and PC2) with centroids of each clade. Green: clade A, blue: clade B, purple: clade C, orange: clade D, pink: clade E, and yellow: clade F

**Fig. 8:**
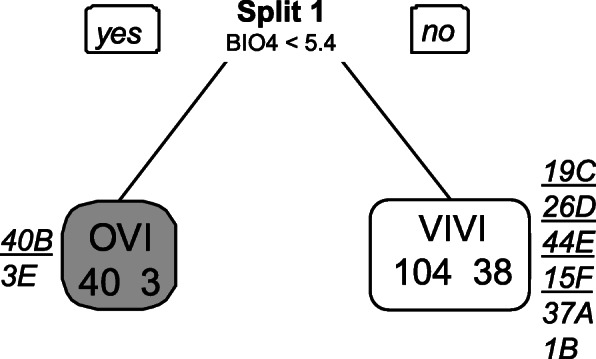
Classification tree for the annual period resulting from recursive partitioning with parity mode (oviparous vs. viviparous) as response variable and the six annual climatic covariates (Appendix S1) as predictors. The variable that decreased the impurity the most is indicated and its threshold value is given (BIO4, units: degrees Celsius). Grey leaves represent the cases classified as oviparous and white leaves represent those classified as viviparous. Numbers within leaves correspond to the number of correctly and incorrectly classified cases (left and right values, respectively). Numbers next to each leave, refer to the number per clade of correctly (underlined) and wrongly classified cases

**Fig. 9:**
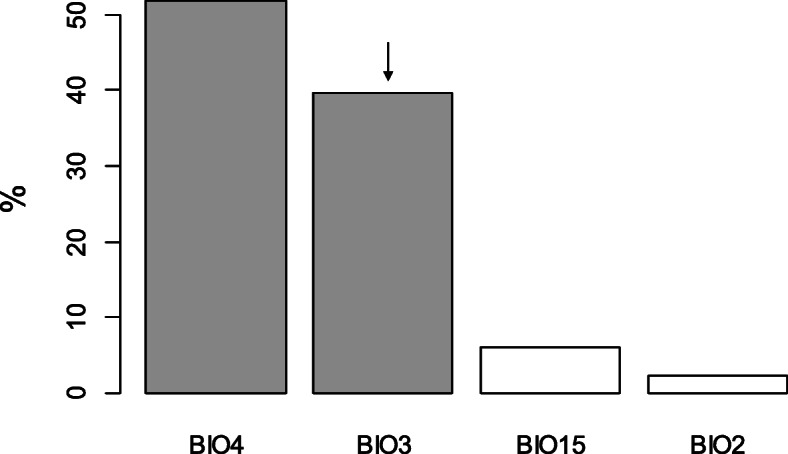
Importance (% of improvement in deviance, y-axis) of the climatic variables (x-axis, for details on their meaning see Appendix S1) in the classification tree of Fig. 8, whose effect is bigger than 1%. Variables whose effect is larger than expected by chance, i.e., those whose importance is larger than 100/(number of variables), are indicated by grey shaded bars. Arrows above significant bars indicate that the climatic variable was negatively correlated with the main variable, i.e., the variable explaining the highest % of the variance. That is, if the main variable’s effect was negative, the effect of the variable indicated with an arrow was positive, or vice versa

As in the analyses based on the reproductive period, principal component analyses of the annual climatic variables rendered two axes (Appendix S3, Figs. 6 and 7), which accounted for a high percentage of the variation (Supporting Information Appendix S3, Fig. 6). The annual analyses (Supporting Information Appendix S3) showed a general low niche overlap between clades, and only the overlap between clades E and F exhibited significantly higher *D*_*obs*_ than expected by chance (Table 3). Moreover, clades exhibited non-equivalent niches in 9 out of 15 comparisons (Table 3). The classification tree showed a high correct classification rate (77.8%), which was similar to that of the analyses based on the reproductive period. Clades C, D, and F were correctly classified, while the entire clade A was misclassified (Fig. 8). In contrast to the analyses based on the reproductive period, viviparous populations occupied locations with higher temperature seasonality (BIO4) than oviparous populations (Fig. 9).

**Table 3:** Results of the niche similarity and equivalency test for the annual period. Below the diagonal, pair-wise niche similarity (*D*_*obs*_) scores are given and asterisks indicate a significant two-tailed niche similary test (* *p* < 0.05; in the significant pair, *D*_*obs*_ was higher than expected by chance). Above the diagonal, significance of pair-wise equivalency tests are shown (* *p* ≤ 0.05, ** *p* ≤ 0.01, NS: not significant; in all significant pairs, *D*_*obs*_ was lower than expected by chance).

Subclade	A	B	C	D	E	F
A	------	**	NS	**	NS	NS
B	0.186	------	**	**	**	*
C	0.660	0.252	------	**	NS	NS
D	0.089	0.000	0.042	------	**	**
E	0.454	0.284	0.421	0.054	------	NS
F	0.511	0.408	0.478	0.025	0.734*	------

## Discussion

This study investigates differences in the climatic niches of all described viviparous and oviparous clades of *Zootoca vivipara* to shed light on the selective forces favouring the evolution and maintenance of viviparity. More specifically, we investigated whether the observed differences in the inhabited climatic niches agree with the predictions of the “cold-climate hypothesis” (CCH [6]), the “maternal manipulation hypothesis” (MMH [7]), and/or the “selfish-mother hypothesis” (SMH [12]). In comparison to previous studies on the evolution of viviparity, this study investigates the evolution of viviparity in a single species, which evolved viviparity relatively recently (≤ 4.4 Mya [29]). Both characteristics potentially increase the likelihood of detecting the drivers of viviparity [22].

Niche equivalence tests showed that in most clade pairs the climatic niches during reproduction were not equivalent and only clades C and F had significantly similar niches (Table 2). Consequently, niche differentiation among clades was strong (see low niche overlap values and many non-equivalent niches; Tables 2). The average climatic niche of clade B (the youngest and oviparous clade [29]) was characterized by the coldest and most variable climate during the reproductive period (Fig. 3c). The viviparous clade D inhabits the warmest average climate (Fig. 3c). Recursive partitioning (Fig. 4) distinguished oviparous from viviparous occurrences with a surprisingly high correct classification rate of 82.7%. Seasonality related variables (BIO3, BIO4, and BIO15) were the most important determinants of parity mode, while variables related with temperature range and climatic averages (BIO2, BIO6, minTmax and meanTmin) had low predictive power (Figs. 4 and 5). Viviparous clades exhibited lower values of BIO4, BIO15, and BIO3 (i.e., three variables related to climatic variability) than oviparous clades (Fig. 4), and thus they inhabit climates with lower variability. These results contrast with the predictions of the “cold-climate hypothesis” (CCH: viviparous populations inhabit colder climates; Table 1; Fig. 1- prediction CCP_1_ [6]). The results also contrast with the “maternal manipulation hypothesis” (MMH), which predicts that viviparous populations preferentially inhabit locations with less favourable climates, e.g. habitats with more variable temperature (Fig. 1- prediction MMP_3_) and/or with higher (Fig. 1- prediction MMP_2_) or lower average temperature (Table 1; Fig. 1- prediction MMP_1_ [9]). However, the results are in line with the prediction from the “selfish-mother hypothesis” (SMH: viviparous populations inhabit less risky habitats, e.g. on average less variable climates; Table 1; Fig. 1- prediction SMP_1_ [12]). Riskier habitats, e.g. climates with higher climatic variability, lead to prolonged gestation [10, 11, 35] and more oxidative damage in mothers [36], and small temperature differences can extend embryonic development by many weeks [11]. Prolonged gestation also increases the female’s exposure to predation [e.g, 30, 31] and viviparous females will therefore favour their own fitness over that of their offspring. Habitats with lower climatic variability are less risky for viviparous females, since the embryonic development is faster and the risk to invest a lot of energy into reproduction without success is lower compared to more variable habitats. Moreover, less variable temperatures during offspring development lead to offspring with higher stamina and increased exploratory behaviour [11], both being positive for the neonate [11]. This suggests that less variable habitats are beneficial for viviparous females and also for their offspring, which points to an adaptive advantage of viviparity.

The higher climatic variability prevailing during the reproductive period in populations of oviparous common lizards may explain why viviparous lizards cannot expand their distributions southwards. In other words, why they cannot colonize habitat inhabited by clade A or B [29]. In contrast, our analyses suggest that oviparous lizards might be able to live in less risky climates that exhibit lower climatic variability. Consequently, population mixing at the suture zone of viviparous and oviparous populations should be frequent. However, recent genetic evidence shows that introgression among viviparous and oviparous lizards is generally low [1, 37], potentially due to reinforcement [1] and higher maternal fitness of viviparous, compared to oviparous females [38]. Moreover, in a contact zone oviparous *Z. vivipara* had shorter telomere length than viviparous *Z. vivipara* [39] and telomer length is negatively linked with extinction risk [16], suggesting that viviparous *Z. vivipara* may have an additional advantage when both reproductive modes are in contact. The finding that viviparous *Z. vivipara* inhabit less variable habitats during reproduction is thus congruent with the idea that viviparity provided an adaptive advantage over oviparity in less risky habitats.

Clade A inhabits the Southern limit of *Z. vivipara’s* distribution and has been suggested to be the most vulnerable clade to climate change [40]. Northward movements of clade A are hindered by the Alps’ main ridge, which consists of insuperable high mountains and perpetual ice over large parts of clade A’s northern distribution limit. Moreover, in the Eastern Alps, northward movements are limited by the presence of other clades (clades: C, E, F [1]). The insuperable northern limit and the fact that clade A exhibits the highest misclassification rate (Figs. 4 and 8), suggest that geographic confinement exists in clade A. This confinement may have favoured a faster change of clade A’s ecological niche compared to the other clades, which may additionally complicate the detection of the drivers of the evolution of viviparity.

The correct classification rate of the classification tree including the reproductive period was higher than that including the annual period (+ 5%; Figs. 4 and 8). The former tree included 4 while the latter tree included only 2 leaves, and in both classification trees the seasonality of temperature (BIO4) was the most important variable in the first split (Figs. 4 and 8). However, during the reproductive period (May–July) lower seasonality of temperature was associated with viviparity, while lower seasonality of temperature during the entire year was associated with oviparity. This shows that studies based on annual climates do not necessarily provide the same results as those based on climates prevailing during the reproductive period, and the results based on one or the other data may not be qualitatively the same. This result contrasts to earlier findings [22], and it stresses that the three hypotheses about the evolution of viviparity need to be tested considering the reproductive period.

## Conclusions

In summary, here we investigated which hypotheses best explain the evolution and maintenance of viviparity at the intraspecific level of *Zootoca vivipara*. The currently inhabited climatic niche of viviparous and oviparous *Z. vivipara* is best explained by the SMH [12], while the predictions of other hypotheses (Table 1, Fig. 1) are incongruent with the currently inhabited climatic niches. Our findings can explain why vivparity may have evolved and why both parity modes are maintained. They suggest that viviparity may have an adaptive advantage over oviparity in less risk climatic conditions (prevailing during the reproductive period) and that viviparous lizards may not be able to invade habitats that are currently occupied by oviparous congeners. This clearly contrasts to previous claims [22, 23, 27] and it suggests that taxa with more recently evolved viviparity may increase the likelihood of detecting the drivers of its evolution. These findings have important implications for the study and understanding of the evolution of reproductive modes in vertebrates, and together with the results of other studies [22, 23], they suggest that in many taxa the evolution of viviparity may have happened too long ago, to detect the ecological drivers that promoted its evolution and success.

## Methods

### Used samples

The European common lizard *Zootoca vivipara* (Lichtenstein, 1823) exhibits two reproductive modes and it consists of six genetic lineages (clades A-F) that inhabit large parts of Eurasia (Supporting Information Appendix S4; Fig. 1; and [29]). Two clades (clades A and B) are oviparous, and four clades are viviparous (C-F [29]). Clade A inhabits the Southern Alps (Northern Italy, Slovenia and Southern Austria) and is the oldest linage (divergence form the other clades: 4.4 Mya; 95% CI = 4.2–2.6 Mya [29]). Clade B inhabits Southern France and Northwest Iberia and diverged 2.0 (1.6–2.41 95% CI) Mya [29]. Clade C and F inhabit Austria, clade D, the Carpathians, North and East Eurasia, and clade E, Eastern and Western Europe and the Southern Balkan [29]. These clades diverged between 2.0 (1.6–2.4) and 2.2 (1.8–2.6) Mya [29]. *Zootoca vivipara* is the terrestrial reptile with the world’s widest and the farthest north distribution [28]. It inhabits temperate, boreal, alpine, Atlantic and continental climates [29] and altitudes from sea level up to over 2400 m above sea level. It passes the winter in hibernacula 2–13 cm below ground [41, 42], where minimum winter temperatures are not lower than a few degrees below zero degrees Celsius [41]. Female common lizards emerge from hibernation in early spring (from March onwards [43]). Mating happens right after emergence [43, 44] and in oviparous populations, egg laying happens on average 1 month after mating. In oviparous populations, females frequently produce two clutches per year, which are laid between May and July [45]. Time of gravidity (from ovulation to oviposition) is temperature dependent [11] and lasts between 25 and 40 days [11]. In viviparous females, the gestation period lasts on average 2 month and parturition of soft-shelled eggs containing fully developed offspring occurs between beginning of June and the end of July [19, 46, 47]. Offspring hatch within one day and are thereafter autonomous, corresponding to ovoviviparous reproduction [48]. In viviparous *Z. vivipara*, the duration of the gestation is not determined by an embryonic signal [19]. Gestation time depends on temperature [11, 49], and females may abort their eggs, and if they do not lay their eggs they may die (personal observations in viviparous and oviparous clades). Moreover, inadequate temperature and humidity regimes during gestation and incubation inevitably lead to deleterious effects on embryos [11, 36].

### Characterization and comparison of climatic niches

For this study we used coordinates from 185 *Z. vivipara* populations from [29], hereafter referred to ‘occurrence records’, belonging to all major *Z. vivipara* lineages (clades A-F) and covering the majority of the known natural Eurasian distribution (Eurasia; Supporting Information Appendix S4; Fig. 1). The environmental PCA (PCA-env) method proposed by Broennimann et al. [34] was used to characterize and compare the realized climatic niches of the *Z. vivipara* clades, since this method performs better than other techniques [34]. The convex hull of the above mentioned 185 *Z. vivipara* populations was considered as the extent of the analyses, since within this area it is reasonable to assume that the species was in contact with the prevailing environmental conditions [50, 51]. This method reduces the amount of locations with non-informative absences [52], a factor shown to importantly affect overlap metrics [53]. Two types of analyses were conducted that tested for climatic differences among clades (1) during the entire year, and (2) during the reproductive period (May–July). Six non-redundant climatic variables related to average climate, diurnal and seasonal variability were considered for the analyses of the annual, and seven variables were considered for the analyses of the reproductive period (Supporting Information Appendix S1). For the correspondence of the two sets of variables see Supporting Information Appendix S1 and for the justification of their use see Supporting Information Appendix S2. All variables were obtained from the Worldclim database [54] at a resolution of 30-arc sec (0.0083 degrees) and monthly Worldclim data was used to derive the different climatic variables for the reproductive period (Supporting Information Appendix S1, S2).

First, 20′000 pixels (0.19% of all pixels) were randomly selected within the area delimited by the convex hull, and a principal component analysis (PCA) was conducted on the environmental variables within the randomly selected pixels and those with occurrence records (20′000 + 185 = 20′185 pixels). Second, a grid of 100 × 100 cells was laid over the ordination space delimited by the two first PCA axes, and a kernel density function was used to create occurrence density plots for each clade. Third, overlaps in occurrence density between two clades were calculated for each pair of clades using the Schoener’s *D* metric (*D*_*obs*_), since this metric performs better than other overlap indexes [53]. Forth, tests of niche similarity and equivalency were performed according to Warren et al. [55] and Broennimann et al. [34]. Briefly, for the similarity tests, for each pair of clades the occurrence density surface of both clades was randomly shifted in the ordination space and *D* was computed (*D*_*sim*_ [56]). This procedure was repeated 1000 times to generate the distribution of *D*_*sim*_, and to test whether *D*_*obs*_ significantly differed from random expectation using a two-tailed test. In the case of the equivalency test, the occurrences of a pair of clades were randomly re-assigned to each clade and the niche overlap (*D*_*sim*_) was calculated. For each pair of clades this procedure was repeated 1000 times to obtain the distribution of *D*_*sim*_ and to test whether *D*_*obs*_ is significantly smaller than expected by chance. The analyses were performed with the ecospat package [56] for R [57].

### Discrimination between parity modes

Recursive partitioning was performed to identify the main variables discriminating between occurrence records of oviparous and viviparous specimens. Classification trees [58] were run in R using the rpart package [59] with parity mode (oviparous vs. viviparous) as binomial response variable and the environmental variables as covariates. First, a tree was built using the Gini coefficient as impurity measure. The complexity parameter was set to zero, the maximum number of surrogate splits to [number of covariates minus one], and a 10-fold cross-validation was used to estimate the relative error. This tree was pruned using the 1-[Standard Error] criterion to get the final parsimonious tree [58]. The importance of each environmental variable across the entire tree and in each split was measured by the decrease in impurity when using the covariate as primary or surrogate variable (see [60] for further details). Again, these analyses were run for the annual as well as the reproductive period (see Supporting Information Appendix S1, S2).

## Data Availability

Used coordinates stem from Horreo et al. [29] and climatic data stems from the Worldclim database [54].

## Appendix S1.- Climatic variables (Worldclim database [54]) used to test for differences in the climatic niche among parity modes. Used abbreviations and variable type (climatic averages, seasonal variability, and diurnal variability), as well as the time span for which the variables were used are given. For justification of their use see Appendix S2

**Table:**

Variable	Abbreviation	Variable type	Time span of models	
			annual	reproductive period
mean diurnal temperature range	BIO2	diurnal variability	X	X
isothermality^*^	BIO3	diurnal variability	X	X
temperature seasonality (standard deviation)^#^	BIO4	seasonal variability	X	X
maximum temperature of warmest month	BIO5	average	X	
minimum temperature of coldest month	BIO6	average		X
mean temperature of warmest quarter	BIO10	average	X	
precipitation seasonality (standard deviation)	BIO15	seasonal variability	X	X
minimum of the monthly maximum temperature	minTmax	average		X
mean of the monthly minimum temperature	meanTmin	average		X

^*^Isothermality corresponds to BIO2/BIO7 (×100) and thus is a variance measure, with values close to 100 indicating that the average diurnal temperature range is the same as than the annual temperature range (BIO7 = BIO5-BIO6) and values smaller than 100 indicating that the average diurnal temperature range is smaller than the annual temperature range

^#^temperature seasonality corresponds to the standard deviation of the monthly average temperature (× 100)

## **Appendix S2.-** Correspondence and justification of the used climatic variables in the annual and reproductive period models

To obtain comparable results between annual and reproductive period analyses, variables which describe similar climatic patterns were used in both analyses (Appendix S1). BIO2, BIO3, BIO4, and BIO15 were used for the annual analyses as well as the reproductive period. BIO6, minTmax and meanTmin were used for the analyses based on the reproductive period. For the same reason BIO5 and BIO10 were used for the analyses based on the entire year. BIO6, minTmax, and meanTmin were not used for the annual analyses since on the Northern Hemisphere minimum temperatures during the coldest month and minimum of the monthly maximum temperature correspond to the hibernation period, which *Z. vivipara* spends in hibernacula where minimum temperatures are not lower than a few degrees below zero [41]. Thus, in the annual models BIO6 does not reflect the climatic conditions to which *Z. vivipara* is exposed. For several reasons, the remaining Bioclimatic variables were not included in any of the models: because they were not relevant for the climatic hypotheses explaining the evolution of viviparity given that they do not reflect the climatic conditions to which *Z. vivipara* is exposed (BIO1, BIO11, BIO12), because of the non-indpendency of certain variables (e.g. BIO7 = BIO5 – BIO6), and because it was not possible to calculate them for the reproductive period (BIO8, BIO9, BIO16-BIO19). Moreover, preliminary analyses including all 19 Bioclimatic variables showed that in our annual dataset the not used variables were redundant: the results including all 19 Bioclimatic variables were qualitatively the same as those presented here, namely, BIO4 was the response variable that decreased the impurty the most and oviparous populations exhibited smaller BIO4 (as in Fig. 8). Moreover, BIO2, BIO3, BIO15 were not of high importance in both annual datasets.

## **Appendix S3.-** results of the annual analyses

Principal component analyses of the climatic variables including the entire year rendered two axes, which accounted for 75.4% of the variation (Fig. 6). The first axis explained 44.1% of the variation. It was positively related with the seasonality of temperature and precipitation (BIO4 and BIO15), and negatively with the other four variables (Fig. 7a). Temperature and precipitation seasonality (BIO4, BIO15) exhibited the lowest loadings, while the other variables exhibited medium loadings (Fig. 7a). The second axis, explained 31.3% of the variation (Fig. 6), and it mainly represents isothermality (BIO3) and temperature seasonality (BIO4; Fig.7b). On average, clade B occupies areas with the highest isothermality and the lowest temperature seasonality, whereas clade D inhabits areas with the lowest isothermality and highest temperature seasonality (Fig. 7c).

Niche similarity tests (Table 3) showed that only clades E and F exhibit significantly higher *D*_*obs*_ than expected by chance, and only clades E-F and A-C exhibit a high overlap (*D*_*obs*_ > 0.6 [34]). In 9 out of 15 comparisons, clades exhibited non-equivalent niches (Table 3).

Cross-validation points to a final tree of two leaves (one split) and a correct classification rate of 77.8% (Fig. 8). 40 out of 78 oviparous records (51.3%) and 104 out of 107 viviparous records (97.2%) were correctly predicted. 40 out of 41 occurrences of clade B (97.6%), 44 out of 47 occurrences of clade E (93.6%), and all occurrences of clade C, D and F were correctly classified. All occurrences of clade A were misclassified (Fig. 8). Variability related variables were the most influential factors (Fig. 9), namely temperature seasonality (BIO4) followed by isothermality (BIO3). Viviparous populations exhibited higher values than oviparous populations (Fig. 8), thus they inhabit areas with higher temperature seasonality and lower relative diurnal temperature variability (Fig. 9).

## **Appendix S4.-***Zootoca vivipara* clade, clade distribution (Fig. 3), sample name, and geographical coordinates of the 185 populations employed in this study

**Table:**

Clade	Sample	Longitude	Latitude
A	OI25 C	13.31005	46.54801
A	OI13 A	13.22471	46.4583
A	OI17A	12.75377	46.5768
A	ZE7	16.4	46.416667
A	ZZ5	14.216667	46.466667
A	OI18 A	10.58579	45.73611
A	OI8 B	11.09293	45.10046
A	OI14 A	12.40341	46.08045
A	OI7 A	13.02966	46.37538
A	ZF50	12.5	46.25
A	OI12 A	13.4835	46.55114
A	ZF51	12.616667	46.4
A	ZZ1–2	14.533333	46.533333
A	OI34 A	11.79626	45.86797
A	OI33 A	8.10722	45.92444
A	HK1	14.966667	46.933333
A	TV5–2	14.516667	47.016667
A	OSL7 A	14.536	45.958
A	OI32 A	11.31	45.9975
A	HK3	14.916667	46.883333
A	VS9 A	9.1206	46.15386
A	Zu9	14.426426	47.018797
A	OSL6 A	14.589	46.425
A	ZZ3	14.766667	46.533333
A	ZZ4–2	14.766667	46.533333
A	OSL4 A	15.614	46.494
A	ZU2–2	14.8	46.933333
A	OI39 A	12.11666	46.78333
A	ZF29	13	45.916667
A	OI40 A	9.86666	45.98333
A	ZE2	14.416667	45.583333
A	ZF43	12.783333	46.316667
A	ZU5	14.183333	46.75
A	OI1 A	8.7	45.784
A	OI27 A	9.41666	45.9
A	OAU1 A	13.24721	46.70479
A	ZZ2	14.566667	46.516667
A	OSL5 A	13.758	46.491
B	691	−1.132169	43.044703
B	692	−1.132169	43.044703
B	OF45A	−0.66954	43.00468
B	BUG1	−0.6383	43.1361
B	09–585	−3.700989	43.117228
B	CS_1200	−0.549079	42.780883
B	CS_1201	− 0.549079	42.780883
B	CS_1202	−0.549079	42.780883
B	CS_1203	−0.549079	42.780883
B	CS_1204	−0.549079	42.780883
B	PF_14_158	−0.397638	42.785724
B	ETH2	−1.017	43.2437
B	CS_527	−0.405052	42.795626
B	CS_526	−0.405052	42.795626
B	CS_531	−0.405052	42.795626
B	OF33C	−0.42331	42.89696
B	PF136	−1.319452	43.021068
B	OF25	−1.07265	43.04206
B	AG09201	−1.80387	43.307327
B	ISA 3	−0.797	43.031
B	OF39 A	−0.64748	42.90117
B	Lizaso_014	−1.675353	42.960353
B	OF41A	−0.54698	42.80262
B	OF31 B	−0.40334	42.7947
B	RC21	−1.314	43.023
B	RHU1	−1.62798	43.31585
B	Lizaso_016	−1.675353	42.960353
B	Lizaso_017	−1.675353	42.960353
B	Lizaso_018	−1.675353	42.960353
B	OE8 B	−6.914	42.866
B	09–503	−7.537261	43.466781
B	09–532	−6.230381	43.001131
B	09–505	−7.537261	43.466781
B	PF-15-109	0.760853	42.627672
B	PF-15-134	0.277289	42.677551
B	OF36A	−0.34547	42.97254
B	BLA3	−0.5492	43.1162
B	PF-15-098	0.997253	42.654031
B	PF-15-100	0.997253	42.654031
B	CAP1	−0.88997	44.21747
B	CET1	−0.5806	42.9351
B	PF-15-125	0.326901	42.94392
B	PF-15-126	0.326901	42.94392
B	PF_14_159	−0.397638	42.785724
B	PF-15-001	2.076389	42.514553
B	PF-15-003	2.076389	42.514553
B	EST 1	−0.22923	42.9098
B	HOU 1	−0.4357	42.9113
B	PF-15-123	0.773036	42.9027
B	Lizaso_015	−1.675353	42.960353
B	OF29 A	−1.294	43.506
B	OF28A	0.158	43.055
B	OF30A	1.980467	42.859806
B	PF_14_234	0.954714	42.715994
B	PF_14_236	0.954714	42.715994
B	PF_14_228	0.954714	42.715994
B	OF27A	−0.727	44.916
B	PF-15-075	1.675611	42.597092
B	SS1 1	−0.396	42.8944
B	PF-15-110	0.760853	42.627672
B	PF-15-135	0.277289	42.677551
B	PF-15-124	0.773036	42.9027
B	PF-15-078	1.675611	42.597092
C	ZL7–2	16.083333	47.533333
C	VAU7 A	15.81342	47.65872
C	ZX1	14.616667	47.566667
C	ZN1	16.8	47.7
C	NZ10	15.033333	47.733333
C	NZ11	15.033333	47.733333
C	NZ14	15.066667	47.083333
C	ZL2–2	16.033333	47.5
C	ZL3–2	16.033333	47.5
C	ZM2	16.45	48.016667
C	VD9–2	14.05	47.15
C	NZ17	15.15	47.766667
C	NZ18	15.15	47.766667
C	VD3–2	14.2	47.4
C	ZX5–2	14.95	47.516667
C	ZX4	14.95	47.516667
C	ZT4	13.583333	47.45
C	ZL6–2	15.716667	47.716667
C	ZL10	15.833333	47.783333
C	VAU6 A	15.851	47.61803
C	VD8	13.933333	47.316667
C	ZL4	15.366667	47.383333
C	ZL5–2	15.366667	47.383333
C	VAU2 B	16.865	47.924
D	ACLV14–26	24.478472	57.112853
D	VR21A	87.25	51.8
D	VRO1 B	22.333	46.197
D	VRO4 A	24.951	47.474
D	VRO4 B	24.951	47.474
D	VH5 E	22.26628	47.76922
D	VR32 B	38.389	56.013
D	VU17 A	33.5	52.33
D	VU18 A	24.229	47.901
D	VRO2 A	24.917	46.66
D	VU19 A	36.583	49.8
D	VH8 A	22.50961	48.01079
D	VBR1 A	23.909	53.911
D	VRO5 D	23.197	45.655
D	VR23 A	29.837	61.154
D	F84	25.749483	62.175583
D	VR38 B	89.26	51.469
D	VR9A	30.349	59.451
D	VRO3 A	22.346	47.314
D	VU20 A	37.352	47.638
D	FO86	25.82115	65.030733
D	S26	22.836517	66.3806
D	VR45 A	48.933	52.283
D	VR26 B	33.823	66.338
D	VR34 C	43.034722	56.039722
D	VR16 A	99.999989	48.88
D	VR39 A	41.458	53.19
D	VR15 A	46.68	55.02
D	VR27 A	28.768	58.434
D	VR24 A	53.09	56.935
E	VI26 A	13.2931	46.55386
E	ZT2	13.95	47.766667
E	VP3	18.2	59.266667
E	VF27 A	3.855	49.905
E	CB12	19.413	43.013
E	VF20 A	6.16917	46.83505
E	VSK3 A	22.087	48.452
E	VF19 A	6.119	45.692
E	vg2	6.55	51.05
E	VS10 A	9.93056	46.78333
E	ZJ1	12.9	48.083333
E	VT2 A	22.65	48.583
E	VU10 A	22.65	48.583
E	VSU3 B	12.309	57.373
E	VAU5 A	12.94962	46.61831
E	ZV1–2	15.15	48.85
E	ZV3	15.15	48.85
E	ZX7	14.466667	47.683333
E	ZX6	14.466667	47.683333
E	VS7 A	8.483	46.733
E	VBE9 B	13.87651	46.91384
E	VU12 A	23.231	48.429
E	VU11 A	23.241	48.839
E	VSK4 A	22.274	48.962
E	VYU2 F	20.685	43.461
E	VP3 D	21.417	53.714
E	VI19 A	13.14621	46.57559
E	VF16 C	3.875	44.385
E	VG1	5.832	51.759
E	VU9 A	22.762	48.619
E	VU14 A	22.762	48.619
E	VB10	23.125	43.122
E	SJ13	23.416667	41.066667
E	VI18 A	13.27794	46.55907
E	VT1	17.989	49.876
E	VI42 A	7.039	44.88416
E	VS5 A	6.183	46.533
E	VSU2 A	16.687	56.703
E	VR6 A	20.848	55.172
E	ZF46	12.7	46.483333
E	ZO1	12.6	47.216667
E	VI39 A	12.95737	46.51414
E	VP1 A	15.539	50.827
E	VH2 B	22.53188	48.12641
E	ZV4	15.066667	48.866667
E	ZV5–2	15.066667	48.866667
E	VI38 A	10.58777	46.26388
E	VI44 A	10.95	46.21666
E	VP2 B	22.654	49.098
E	VI43 A	10.68333	46.48333
E	VI21 A	11.76603	46.42007
E	VP1	12.633333	51.316667
F	WE-11	13.383333	46.866667
F	WS11	12.466667	46.816667
F	VAU4 D	13.14853	46.76571
F	VAU4 A	13.14853	46.76571
F	VH7 A	19.26977	47.23372
F	VH7 C	19.26977	47.23372
F	ZY1–2	14.916667	47.1
F	ZY2	14.916667	47.1
F	ZY4	15	47.016667
F	VAU8 A	14.93428	46.78386
F	VU3	19.35	46.8
F	VU-2	19.35	46.8
F	VU4–2	19.35	46.8
F	ZV7	14.733333	46.933333
F	ZU8	14.733333	46.933333
F	VH1	14.95	46.48
F	VH2	14.95	46.48
F	WE12	13.25	46.983333
F	VD1	14.15	47.1
F	VD2	14.15	47.1
F	VH4 D	19.22784	47.27035
F	VH4 E	19.22784	47.27035
F	ZU10	14.783333	46.95
F	ZY3	15.283333	47.333333

## Abbreviations

*σ*^2^: (variance)
$$ \overline{X} $$: (average)
BIO2: Mean diurnal temperature range
BIO3: Isothermality
BIO4: Temperature seasonality
BIO5: Maximum temperature of warmest month
BIO6: Minimum temperature of coldest month
BIO10: Mean temperature of warmest quarter
BIO15: Precipitation seasonality
CCH: Cold climate hypothesis
CCP_1_: Cold climate prediction 1
CI: Confidence interval
D_obs_: Observed overlap in occurrence density between two clades
D_sim_: Simulated overlap in occurrence density between two clades
meanTmin: mean of the monthly minimum temperature
minTmax: Minimum of the monthly maximum temperature
OVI: Oviparous
Mya: Millon years ago
MMH: Maternal manipulation hypothesis
MMP_1_: Maternal manipulation prediction 1
PCA-env: Environmental principal components, calibrated on the entire environmental space
PC1: Principal component one
PC2: Principal component two
SMH: Selfish-mother hypothesis
SMP_1_: Selfish-mother prediction 1
VIVI: Viviparous
